# Handing off hope: transition of care in pediatric surgery

**DOI:** 10.1007/s00464-025-12561-z

**Published:** 2026-03-02

**Authors:** Claire M. Wunker, Bethany J. Slater, Rodrigo Gerardo, Lori Gurien, Carroll M. Harmon, Janey S. A. Pratt, Alessandra C. Gasior

**Affiliations:** 1 Department of Surgery, C.S. Mott Children’s Hospital, University of Michigan, Ann Arbor, MI USA; 2 Department of Surgery, Mount Sinai Kravis Children’s Hospital, New York, NY USA; 3 Department of Surgery, University Hospitals, Ravenna, OH USA; 4 Department of Surgery, Nemours Children’s Health, Jacksonville, FL USA; 5 Department of Surgery, University at Buffalo, Buffalo, NY USA; 6 Department of Surgery, Lucille Packard Children’s Hospital, Stanford Children’s Health, Palo Alto, CA USA; 7 Department of Surgery, The Ohio State University Wexner Medical Center, 410 W. 10th Avenue, Columbus, OH USA

**Keywords:** Pediatric surgery, Transition of care, Congenital malformations, Bariatric surgery, Inflammatory bowel disease, Gastrointestinal malignancy

## Abstract

**Background:**

The importance of transitioning care from pediatric to adult practitioners is an often overlooked aspect of chronic disease. The benefits of a planned transition in health care are that patients will learn valuable life-long lessons on healthcare maintenance, when and how to seek medical attention for new issues that may arise, and improvements on their overall well-being. The purpose of this SAGES White Paper was to summarize the available knowledge for several pediatric surgical conditions to aid in transition of care for this patient population.

**Methods:**

The members of the SAGES Pediatric Surgery Committee elected to produce this White Paper. The group chose to focus on several important gastrointestinal diseases that may require lifelong care: tracheoesophageal fistula, duodenal atresia, anorectal malformations, childhood obesity, and gastrointestinal malignancies. For each disease process a summary of long term issues facing these patients, stakeholders involved, and follow up recommendations if required were identified.

**Results:**

Each disease process has its own unique set of long-term issues as well as multidisciplinary stakeholders and need for follow-up. However, individualized care is needed based on each patient’s unique needs. To facilitate consistent transfer of care standardization is needed for surgical diseases. Key aspects of standardization include identifying a multidisciplinary team, working towards consistent quality improvement, and implementation of policy guided processes with individual treatment plans.

**Conclusion:**

Continued work in standardizing transition of care is required for optimal treatment of this complex patient population.

**Supplementary Information:**

The online version contains supplementary material available at 10.1007/s00464-025-12561-z.

The role of transitional care in pediatric surgery has not been a significant focus until recently. In pediatric settings, care delivery is uniquely structured compared to adult medicine. Pediatric surgeons often act as the central providers for children with complex surgical needs—frequently functioning much like a primary care provider, coordinating and overseeing multiple aspects of the child’s treatment and health maintenance and following them longitudinally. This contrasts sharply with adult surgical care, where multidisciplinary teams tend to be more prevalent and primary care providers often function in management roles. As a result, pediatric patients develop close, longitudinal relationships with their surgical team, making the eventual transition to adult care both complex and emotionally significant. The benefits of a planned transition in health care are that patients will learn lifelong valuable lessons on healthcare maintenance, when and how to seek medical attention for new issues that may arise, and improvements on their overall well-being [1].

We have learned from our adult patients that a lack of transitional care can cause increased healthcare utilization costs and poor long-term consequences, including physical and mental health implications. However, significant barriers impede the transition from pediatric to adult care. Fragmented systems of care often leave patients and families navigating unclear pathways, and adult providers may lack the specialized knowledge or comfort required to manage the sequelae of congenital or childhood-onset conditions. A recent survey of young patients with congenital bowel and bladder conditions showed that less than half had transitioned into adult care [2]. Another survey study demonstrated that of only 54% of participants who had transitioned to adult care, 67% stated there was no discussion about transition prior to their transition into adult care and 71% were not given any recommendation for which provider to transition [3]. Generally, patients respond that the lack of support and limited knowledge by adult providers is the most difficult part of transitional care [4]. When adult surgeons were asked about perceived barriers to transition of care, the lack of a structured program for transitional care at their institution, lack of understanding of the complex post-surgical anatomy, and difficulty identifying qualified adult surgeons within a reasonable access area were cited [5]. Transition of care is a relatively standardized practice in many congenital pediatric diagnoses including congenital cardiac disease, diabetes, cystic fibrosis, and transplantation [6, 7]. These are often thoroughly planned, well-designed, and utilize a multidisciplinary approach [8]. It is important that this is seen as a process, not a singular event, and to aid in this, online toolkits exist [9]. These provide useful models for possible improvements across specialties, highlighting the critical need for structured programs, readiness assessments, and ongoing education for both patients and providers.

Overall, the data remain lacking, with small series and single-center reporting, but this paper seeks to consolidate the literature by summarizing the key needs of common conditions that require transition from pediatric to adult surgical care. We emphasize not only what the adult provider should know about the natural history, potential complications, and psychosocial aspects of these conditions, but also advocate for system-level recommendations to optimize the transitional process. By compiling best practices—such as starting transition discussions early, developing comprehensive care plans, fostering communication, and maintaining psychosocial support—this work aims to inform future guidelines and policy development. Ultimately, a planned and patient-centered transition through a policy-designed process offers lifelong benefits: It empowers patients to manage their health, reduces care gaps, and promotes sustained well-being into adulthood.

## How to transition patients

One of the most important aspects of transitional care is having a policy-defined process in place at each healthcare facility and discussing this with parents and patients at their early visits with their surgical team. This process should be introduced years before adulthood and is explained with each visit thereafter until the patient is transitioned to the adult practice. Annual readiness assessments can be given to the patient in order to provide a self-care skill assessment and use this as a guideline for deficiency identification and tailored education. It is also important to provide an annual survey to the caregiver or parent as the patient slowly transitions into a more autonomous role. (Supplemental Documents 1 and 2) Additionally, a multidisciplinary transferring team for all aspects of medical care for the patient should be identified on both the pediatric and adult sides.

When the patient and parent surveys demonstrate that the patient is autonomously ready to transition to adult health care, it is important that the final decision to transition involves all stakeholders and is completed during a period of medical stability. Most importantly, the patient and parent must both be in agreement to transition. It must also be acknowledged that some patients may never cognitively meet the criteria for autonomous medical decision making; therefore, the decision to transition to adult care should take place with the continued assistance and guidance of their medical proxy and caregiver. Patients and families also need genuine and significant closure to longstanding relationships with their pediatric surgical team to help them bridge into the adult healthcare system as the medical trauma of chronic conditions may be weighty [10].

There are many models that can exist on which the providers a patient transitions to, depending on the institution. For example, a pediatric hospital system that is integrated within an adult hospital system will likely transfer a patient to their own adult providers, whereas institutions with a free-standing pediatric hospital likely would refer within their academic affiliation. Other practices may have providers who practice both pediatric and adult care or refer to the nearest geographic location with appropriate providers. There is no one size fits all, but the process must be optimized with periodic feedback from the patients who have gone through the process to continuously improve upon the process.

### Best practices for transition

While individuals will require tailored plans for transitioning care, there are several general tenets for a successful implementation.

1. Start early: After completion of treatment while in the surveillance phase, introduce the concept of transition to the patient and family. This will be somewhat dependent upon the patient’s age at diagnosis and is more pressing for older children.
2. Develop a comprehensive plan: Includes a summary of the patient’s medical and surgical history, follow-up schedule, and future care needs. This should be done by the patient’s primary provider with assistance from associated specialists as needed. This is a living document that should be updated throughout treatment and follow-up.
3. Educate patients and families: Emphasize the importance of ongoing care and equip them with knowledge about potential risks and symptoms. Education starts from diagnosis and proceeds long after treatment is complete. Discussions regarding potential long-term complications should be included.
4. Foster communication: Ensure seamless information transfer between pediatric and adult providers. Excellent documentation can aid in discerning treatment histories, durations, surgical procedures, and potential complications.
5. Provide psychosocial support: Address fears, anxieties, and practical concerns about transitioning between providers.
6. Adopt a multidisciplinary approach: Engage all relevant stakeholders to provide coordinated care.

### Esophageal atresia and tracheoesophageal fistula

Esophageal atresia (EA) and tracheoesophageal fistula (TEF) are rare congenital disorders affecting approximately 1:2500–3000 live births [11]. Several congenital anomalies are frequently associated with EA, most commonly cardiac and anorectal. Treatment depends on the anatomic classification as well as the other comorbidities of the patient. However, typically operative intervention with division of the fistula and anastomosis of the esophagus is performed in the first few days of life.

Due to improved intensive care and surgical techniques, survival rates in high-income countries have dramatically improved over the last couple of decades to greater than 90% [12]. As such, many EA/TEF patients need a transition of care in adolescence and adulthood. However, a survey of the European Reference Network on rare Inherited and Congenital Anomalies (ERNICA) centers found that only 55% of all those centers reported the presence of a transition pathway [13]. Thus, attention to the transition of quality care for EA/TEF patients through dedicated multidisciplinary programs or through increased knowledge of adult providers caring for these patients is extremely important.

#### Long-term issues

Patients that have been treated for EA/TEF have several long-term issues that persist into adolescence and adulthood, including gastrointestinal issues such as gastroesophageal reflux, dysphagia, and risk of esophageal cancer, respiratory problems, musculoskeletal abnormalities, neurologic sequelae, and quality of life (QOL) concerns. A systematic review investigating the prevalence of chronic long-term problems demonstrated that 50% of EA/TEF patients experience dysphagia in the long-term, which is a risk factor for gastroesophageal reflux disease (GERD) and esophagitis [14]. Respiratory problems consist of cough, recurrent infections, and asthma [15]. The musculoskeletal abnormalities, most notably secondary scoliosis, likely depend on the type of repair performed, with a higher incidence after thoracotomies. The Federation of Esophageal Atresia and Tracheo-Esophageal Fistula support groups (EAT) reported on long-term outcomes and quality of life in a large international cohort of patients, including eating ability, GERD, and respiratory problems [16]. This study also highlighted that as patients’ age increased, they were less likely to have a medical provider [16].

#### Stakeholders

These include pediatric, orthopedic, and foregut surgeons; gastroenterologists; pulmonologists; psychologists; neurologists; dietitians; and primary care physicians.

#### Follow-up

Currently, there are no universal esophageal and respiratory follow-up standards for patients with EA; however, the International Network on Oesophageal Atresia (INoEA) has formulated clinical practice guidelines for the care of EA/TEF patients during adolescence and adulthood [17]. Recommendations for follow-up regarding gastrointestinal disorders included visits with a gastroenterologist every two years until the age of 35 and yearly afterward. Endoscopy with biopsies should be performed every five years until the age of 28 and then more frequently after that. Of note, the ESPGHAN/NASPGHAN surveillance guidelines suggest endoscopy with biopsy be performed every 5–10 years with as-needed endoscopies for any new or worsening symptoms [18]. Regarding respiratory concerns, the group suggested the use of pulmonary function tests along with chest CT scan for the detection of bronchiectasis as indicated [17].

### Duodenal atresia

Congenital anomalies of the duodenum present with intestinal obstruction and are usually repaired during the neonatal period. However, patients with these conditions may often require management during later years for either long-term complications after repair as a child or for late diagnosis during adulthood. Most common long-term complications that require surgical intervention include GERD, megaduodenum, peptic ulcers, and adhesive small bowel obstructions [19]. Other long term complications can include anastomotic stricture and gastric outlet obstruction [19]. Although a duodenoduodenostomy is the current preferred repair approach, patients who have previously undergone repair with a gastrojejunostomy or duodenojejunostomy can be affected by blind loop syndrome [20].

#### Long-term issues

The incidence of long-term complications is unclear, but they are likely more common than what has previously been reported in the literature since most studies focus on surgical/endoscopic interventions and long-term medication use rather than patient-reported gastrointestinal symptoms, for which they may not have sought or received treatment [21]. Common complications can include reflux, bowel obstruction, stricture, missed web, or megaduodenum. The need for anti-reflux procedures has decreased with medical management and more extensive use of acid suppressants [22]. Patients with a history of congenital duodenal obstruction who present with signs and symptoms of adhesive small bowel obstruction should be managed in a similar fashion to any patient presenting with a small bowel obstruction and a history of previous surgical intervention. As laparoscopic duodenal atresia repair becomes more common, bowel obstruction from adhesive disease may decrease in frequency [20, 22].

For patients with a history of duodenal atresia who present with symptoms of abdominal pain, early satiety, emesis, halitosis, and/or weight loss, a workup should be performed to assess the anastomotic area to evaluate for stricture, missed web, and/or megaduodenum. Imaging with contrast studies can evaluate the anatomy of the duodenum to help determine the cause of the symptoms [20, 22]. Anastomotic strictures can sometimes be managed with balloon dilation, but otherwise will require surgical revision [22]. There is evidence to suggest that these patients would benefit from being followed into adulthood, especially those with a history of annular pancreas [19]. Adult providers who commit to caring for these patients later in life should have a high level of suspicion for these long-term complications and intervene when indicated.

#### Stakeholders

These include pediatric and adult gastroenterologists, pediatric and hepatobiliary foregut surgeons, and primary care providers.

### Bariatric patients

Childhood obesity represents a chronic disease, with strong heritability that leads to multiple comorbid conditions. Recent Clinical Practice Guidelines from the American Academy of Pediatrics recommend using Obesity Management Medications (OMMs) and Metabolic and Bariatric Surgery (MBS) for the treatment of children with severe forms of childhood obesity. It is clearly important to treat obesity as soon as it starts, even in childhood, since these comorbid conditions can be both life threatening and significantly affect quality of life. Insulin resistance syndrome that progresses to Type 2 Diabetes Mellitus (T2DM), metabolic associated liver steatosis that progresses to steatohepatitis, and sleep apnea are the most common and severe comorbid conditions that develop in the setting of severe obesity in children.

Treating childhood obesity with MBS results in resolution of these comorbid conditions in addition to weight loss and improved quality of life. In the largest study of pediatric patients in the United States, Teen LABS, MBS alone provides sustained weight loss in about 50% of patients while 11% are seen to regain the weight within a few years [23]. Furthermore, 85% and 91% of adolescents will have resolution of their T2DM and sleep apnea, respectively [24].

#### Long-term issues

When a child or adolescent undergoes MBS in a pediatric hospital, it is imperative that they can continue their postoperative care in an adult bariatric surgery center to monitor nutritional deficiencies, weight recurrence, progression or recurrence of comorbid conditions, mental health conditions, and late complications of surgery. Obesity is a lifelong disease and will likely re-occur at times of major metabolic events like pregnancy, menopause, and after injuries or accidents.

Most patients can be followed in medical obesity clinics by physicians who are board certified in obesity medicine. The adjunct use of OMMs makes long-term follow-up in these clinics more important than ever, but it is also important to remember that obesity medicine boards cover bariatric surgery and therefore patients benefit from providers who may be more attentive to post-surgical complications. Patients themselves should be made aware of signs and symptoms of common vitamin deficiencies and other surgical complications to know when to seek care. This is one reason to have an adult metabolic and bariatric surgeon see every patient who has had obesity and bariatric surgery in childhood. Perhaps not on a regular basis if they are in an OM clinic, but at least once to establish a relationship, should surgical complications arise later on in life.

Hart and Eneli showed that using an embedded electronic health registry improved patient retention rates in a bariatric program, allowing for a more effective transition of care [25]. They were also able to demonstrate that the standardization of care and health coaching programs was effective in assisting transition and resulted in improved outcomes. However, these programs were dependent on the staff’s relationship with the patients and were therefore often unsustainable. The most notable finding of their QI project in patient follow-up and transition was the effect of the loss of their bariatric coordinator, which had a significant direct impact on patient follow-up and participation in the postoperative registry, nearly tripling their loss to follow-up. This indicates that the coordination of care is essential for appropriate follow-up.

#### Stakeholders

These include pediatric and adult bariatric surgeons, board-certified obesity medicine physicians, dieticians, and primary care providers.

#### Follow-up

Long-term complications and needs for revisional surgery depend completely on the type of operation that the patient has had. Sleeve gastrectomy patients should be monitored for reflux and are recommended to undergo screening endoscopy prior to transition to an adult program or within 5 years of the sleeve, while gastric bypass patients need to be monitored for abdominal pain (ulcers, stones, or obstructions), gastrointestinal bleeding, and drug/alcohol addiction [26]. Both operations can see weight regain and nutritional deficiencies that require adjunct OMM’s, revisional surgery, and/or vitamin supplement adjustments. Early recognition and treatment of complications are critical to decreasing surgical morbidity.

### Anorectal malformation and hirschsprung disease

Anorectal malformation (ARM) is a congenital colorectal condition where the anus and rectum do not develop properly. It can also involve the urinary or reproductive organs and be associated with other organ system abnormalities, including cardiac, renal, spinal, limb, trachea, and esophagus. Hirschsprung disease (HD) is a congenital condition in which nerve cells (ganglion cells) are absent in parts of the colon, most commonly the rectum and sigmoid colon. The lack of ganglion cells leads to poor peristalsis, constipation, obstruction, and enterocolitis.

#### Long-term issues

During adolescence, 89% of ARM patients develop new concerns that require intervention from multiple specialty teams including fecal and urinary incontinence, constipation, ejaculatory dysfunction, erectile dysfunction, and psychosocial issues, thus decreasing their overall quality of life [27]. More than 30% of ARM patients will continue to have concerns with constipation, fecal incontinence, fertility, and sexual function throughout their lifetime [28–30]. Furthermore, the majority of patients are not well educated on what type of anorectal malformation they had and what their current anatomy entails. In one single-center study of 111 patients, only 29% of adult patients knew their type of anorectal malformation [3].

Post-operative issues for patients with Hirschsprung disease can be broken down into soiling or obstruction. For those patients with soiling, damage to the sphincter should be considered. For those with obstructive symptoms, anatomic or pathologic complications may necessitate a redo–pull through while non-relaxation of the sphincters may require botox injection. Long-term, an estimated 44% of Hirschsprung disease patients will have constipation [1].

Adult female patients with a history of anorectal malformation may require more extensive perinatal workup prior to vaginal birth due to variations in the pelvis and pelvic floor anatomy. Vaginal delivery should be avoided in women with a history of any type of anorectal malformation, relegating those patients to C-section delivery, which should be done with the consultation of a pediatric or colorectal surgeon.

Lastly, the transition to adult care for these patients should include a strong consideration for their psychosocial issues [31]. As patients move toward adulthood, this transition should be gradual, considering the individual’s readiness for change and recruiting the assistance of psychologists not only for mental preparation but also for the empowerment of one’s own health [1]. This can be augmented by the utilization of effective patient education and even engagement in patient support networks that are dedicated to the unique needs of this patient population [31].

#### Stakeholders

These include pediatric and adult gastroenterologists, pediatric and colorectal surgeons, pediatric and adult urologists, pediatric and adult gynecologists, fertility specialists, social workers, and psychiatrists.

### Inflammatory bowel disease

Inflammatory bowel disease (IBD) is a diagnosis that is more frequently seen in adult patients and thus the transition of care requires less outside knowledge from their adult providers. However, pediatric patients with IBD tend to have a more severe clinical phenotype and have a higher risk of malignancy than patients who are diagnosed with IBD in adulthood [32].

#### Long-term issues

The transition period from adolescents to young adulthood is challenging and poses great risk for poor follow-up and medication compliance, which can lead to worsening of disease and antibody formation. A well-established transition program improves relapse, admission rates, and steroid usage [33]. Patients with J-pouches may develop pouch prolapse, fecal incontinence, sexual dysfunction, adhesions, strictures, or pouchitis [34]. Similarly to other patients with previous pelvic surgery, the mode of birth delivery will need to be evaluated for female patients, particularly those with a J-pouch who may need to consider a C-section to preserve sphincter function.

#### Stakeholders

These include adult and pediatric gastroenterologists, pediatric and colorectal surgeons, nutritionists, psychologists, and social workers.

### Gastrointestinal malignancies

Pediatric gastrointestinal (GI) malignancies, though rare, represent a significant health challenge due to their aggressive nature and the intensive treatments required. The most common GI malignancy is lymphoma, although other tumors can include various carcinomas, inflammatory pseudotumors, Peutz-Jegher polyps, neurofibromas, and carcinoids [35]. Advances in therapy have led to improved survival rates, but survivors face numerous long-term challenges. Transitioning these patients to adult care is a pivotal step in ensuring continued health monitoring and addressing residual effects of treatment.

#### Long-term issues

Pediatric GI malignancies require multimodal treatment strategies, including surgery, chemotherapy, and sometimes radiation therapy [35]. As such, long-term consequences are ongoing with up to 65% of patients having chronic health problems. Survivors may also have organ impairment secondary to chemotherapy, radiation, or surgery [36]. The Children’s Oncology Group provides an excellent resource for clinicians regarding long-term survivorship and possible side effects from specific treatments [37].

Survivors are at risk for recurrence or metastasis, post-surgical complications, growth and developmental delays, chronic health conditions, and secondary malignancies [38, 39]. In a large-scale study of pediatric cancer patients, recurrence was the most common contributor to the cause of death, while subsequent cancers, pulmonary, and cardiac dysfunction were also frequent contributors [38]. Routine assessment with physical exams, imaging, and biomarkers is key for detecting recurrence. After the initial follow-up period, patients will often be transitioned back to primary care providers for monitoring, and so the surveillance schedule with routine testing may not be necessary; however, a low threshold for repeat testing should be present.

Long-term effects also include fertility impairment and patients should be referred to specialist providers when trying to conceive [40]. Specifically in GI malignancies, as opposed to blood cancers which are much more common in this population, patients often undergo surgical resection. This can lead to postoperative complications including adhesions, bowel obstructions, or functional impairments. Patients may also require surgery for revision or recurrence. These patients can also be affected by psychosocial and growth delays. The psychosocial challenges after cancer treatment can persist and providing appropriate support to the patient and their family is important for long-term recovery. This can include encouraging appropriate healthcare coverage, clinical supports, healthy behaviors, and health maintenance.

#### Stakeholders

These include pediatric and adult oncologists, pediatric surgeons, surgical oncologists, primary care physicians, dieticians, and psychologists.

#### Follow-up

Follow-up care should be individualized, but in general will include a period of intense follow-up with repeat imaging and laboratory testing at regular intervals. Often this will space out to longer periods as tests continue to be negative for recurrence. Some providers may stop doing regular testing and move only toward routine testing and cancer screening to evaluate for secondary malignancies. Often, many years after diagnosis, visits can be extended based on risk factors and symptomatology.

## Conclusion

The importance of transitioning care from pediatric to adult practitioners is an often overlooked aspect of chronic disease. The benefits of a planned transition can include increased patient engagement, improved healthcare maintenance, and improvement in overall well-being [1]. Standardized transition of care is needed for pediatric surgical diseases including a multidisciplinary team, consistent quality improvement, and policy-guided processes with individual treatment plans. Continued work in this area is required for optimal treatment of this complex patient population.

## Supplementary Information

Below is the link to the electronic supplementary material.

Supplementary material 1 (PDF 511.5 kb)

Supplementary material 2 (PDF 582.6 kb)

## References

[1] Gasior A, Midrio P, Aminoff D, Stanton M (2020) Ongoing care for the patient with an anorectal malfromation; transitioning to adulthood. Semin Pediatr Surg 29:150991. 10.1016/j.sempedsurg.2020.150991 33288136 10.1016/j.sempedsurg.2020.150991

[2] Loftus CJ, Ahn J, Rice-Townsend S, Avansino J, Schmidt J, Hagedorn JC, Wood R, Shnorhavorian M, Fuchs MD, McCracken KA, Hewitt G, Amies-Oelschlager A-ME, Merguerian P, Smith CA (2021) Experiences and attitudes of young adults with congenital bowel and bladder conditions. J Pediatr Urol 17:701.e1-701.e8. 10.1016/j.jpurol.2021.06.014 34217590 10.1016/j.jpurol.2021.06.014

[3] Vargas MC, Wehrli LA, Louiselle A, Ketzer J, Reppucci ML, Juddy-Glossy L, Alaniz VI, Wilcox DT, Wood DN, Peña A, De La Torre L, Bischoff A (2022) Do adult patients with congenital colorectal conditions know their diagnosis? Pediatr Surg Int 38:1723–1728. 10.1007/s00383-022-05220-0 36129533 10.1007/s00383-022-05220-0

[4] van der Bent A, Duggan EM, Fishman LN, Dickie BH (2018) Reality check: what happens when patients with anorectal malformations grow up? A pilot study of medical care transition from the adult patient perspective. J Pediatr Surg 53:1722–1726. 10.1016/j.jpedsurg.2018.02.057 29605261 10.1016/j.jpedsurg.2018.02.057

[5] Ahmad H, Knaus ME, Minneci PC, Wood RJ, Gasior AC (2022) Transition of care barriers in pediatric patients with anorectal malformation. Dis Colon Rectum 65:955–957. 10.1097/DCR.0000000000002368 35535807 10.1097/DCR.0000000000002368

[6] Nagra A, McGinnity PM, Davis N, Salmon AP (2015) Implementing transition: ready steady go. Arch Dis Child Educ Pract Ed 100:313–320. 10.1136/archdischild-2014-307423 26063244 10.1136/archdischild-2014-307423 PMC4680199

[7] Connett GJ, Nagra A (2018) Ready, steady, go - achieving successful transition in cystic fibrosis. Paediatr Respir Rev 27:13–15. 10.1016/j.prrv.2018.05.007 29914748 10.1016/j.prrv.2018.05.007

[8] Weitz M, Heeringa S, Neuhaus TJ, Fehr T, Laube GF (2015) Standardized multilevel transition program: does it affect renal transplant outcome? Pediatr Transplant 19:691–697. 10.1111/petr.12570 26260514 10.1111/petr.12570

[9] Got Transition (n.d.) Resources & research. In: GotTransition.org. https://gottransition.org/resources-and-research/. Accessed 23 Oct 2025

[10] Baumbusch J (2024) Cliff or bridge: breaking up with the paediatric healthcare system. Paediatr Child Health 29:84–86. 10.1093/pch/pxad061 38586492 10.1093/pch/pxad061 PMC10996575

[11] Coran AG, Adzick NS (2012) Pediatric surgery, 7th ed. Elsevier Mosby, Philadelphia, PA

[12] Lopez PJ, Keys C, Pierro A, Drake DP, Kiely EM, Curry JI, Spitz L (2006) Oesophageal atresia: improved outcome in high-risk groups? J Pediatr Surg 41:331–334. 10.1016/j.jpedsurg.2005.11.009 16481246 10.1016/j.jpedsurg.2005.11.009

[13] Durkin N, Pellegrini M, Gorter R, Slater G, Cross KMK, Ure B, Wijnen R, Gottrand F, Eaton S, De Coppi P; ERNICA (2024) Follow-up and transition practices in esophageal atresia: a review of European Reference Network on rare Inherited and Congenital Anomalies (ERNICA) centres and affiliates. Pediatr Surg Int 40:300 39521743 10.1007/s00383-024-05865-z PMC11550284

[14] Connor MJ, Springford LR, Kapetanakis VV, Giuliani S (2015) Esophageal atresia and transitional care–step 1: a systematic review and meta-analysis of the literature to define the prevalence of chronic long-term problems. Am J Surg 209:747–759. 10.1016/j.amjsurg.2014.09.019 25605033 10.1016/j.amjsurg.2014.09.019

[15] Brooks G, Gazzaneo M, Bertozzi M, Riccipetitoni G, Raffaele A (2023) Systematic review of long term follow-up and transitional care in adolescents and adults with esophageal atresia - why is transitional care mandatory? Eur J Pediatr 182:2057–2066. 10.1007/s00431-023-04893-6 36905437 10.1007/s00431-023-04893-6 PMC10175361

[16] Svoboda E, Fruithof J, Widenmann-Grolig A, Slater G, Armand F, Warner B, Eaton S, De Coppi P, Hannon E (2018) A patient led, international study of long term outcomes of esophageal atresia: EAT 1. J Pediatr Surg 53:610–615. 10.1016/j.jpedsurg.2017.05.033 28622972 10.1016/j.jpedsurg.2017.05.033

[17] Krishnan U, Dumont MW, Slater H, Gold BD, Seguy D, Bouin M, Wijnen R, Dall’Oglio L, Costantini M, Koumbourlis AC, Kovesi TA, Rutter MJ, Soma M, Menzies J, Van Malleghem A, Rommel N, Dellenmark-Blom M, Wallace V, Culnane E, Slater G, Gottrand F, Faure C (2023) The International Network on Oesophageal Atresia (INoEA) consensus guidelines on the transition of patients with oesophageal atresia–tracheoesophageal fistula. Nat Rev Gastroenterol Hepatol 20: 735–755 10.1038/s41575-023-00789-w 10.1038/s41575-023-00789-w 37286639

[18] Krishnan U, Mousa H, Dall’Oglio L, Homaira N, Rosen R, Faure C, Gottrand F (2016) ESPGHAN‐NASPGHAN Guidelines for the evaluation and treatment of gastrointestinal and nutritional complications in children with esophageal atresia‐tracheoesophageal fistula. J pediatr gastroenterol nutr 63:550–570. 10.1097/MPG.0000000000001401 10.1097/MPG.0000000000001401 27579697

[19] Escobar MA, Ladd AP, Grosfeld JL, West KW, Rescorla FJ, Scherer LR, Engum SA, Rouse TM, Billmire DF (2004) Duodenal atresia and stenosis: long-term follow-up over 30 years. J Pediatr Surg 39:867–871. 10.1016/j.jpedsurg.2004.02.025 15185215 10.1016/j.jpedsurg.2004.02.025

[20] Lum Min SA, Imam M, Zrinyi A, Shawyer AC, Keijzer R (2023) Post-discharge follow-up of congenital duodenal obstruction patients: a systematic review. Pediatr Surg Int 39:239. 10.1007/s00383-023-05515-w 37490166 10.1007/s00383-023-05515-w

[21] Treider MA, Røkkum H, Sæter T, Bjørnland K (2025) Gastrointestinal quality of life after congenital duodenal obstruction repair: a nationwide long-term follow-up study. J Pediatr Surg 60:161938. 10.1016/j.jpedsurg.2024.161938 39332973 10.1016/j.jpedsurg.2024.161938

[22] Cullis PS, Fouad D, Goldstein AM, Wong KKY, Boonthai A, Lobos P, Pakarinen MP, Losty PD (2024) Major surgical conditions of childhood and their lifelong implications: comprehensive review. BJS Open 8:Zrae028. 10.1093/bjsopen/zrae028 38776252 10.1093/bjsopen/zrae028 PMC11110943

[23] Ryder JR, Jenkins TM, Inge TH, Courcoulas AP, Harmon CM, Helmrath MA, Sisley S, Michalsky MP, Brandt M (2024) Ten-year outcomes following adolescent bariatric surgery. N Engl J Med 391:1656–1658. 10.1056/NEJMc2404054 39476348 10.1056/NEJMc2404054 PMC11753263

[24] Ruiz-Cota P, Bacardí-Gascón M, Jiménez-Cruz A (2019) Long-term outcomes of metabolic and bariatric surgery in adolescents with severe obesity with a follow-up of at least 5 years: a systematic review. Surg Obes Relat Dis 15:133–144. 10.1016/j.soard.2018.10.016 30514669 10.1016/j.soard.2018.10.016

[25] Hart LC, Eneli I (2024) Retention and transition to adult health care in adolescent bariatric surgery. Surg Obes Relat Dis 20:1314–1321. 10.1016/j.soard.2024.06.005 39117559 10.1016/j.soard.2024.06.005

[26] Kassir R, Kassir R, Deparseval B, Bekkar S, Serayssol C, Favre O, Garnier P-P (2019) Routine surveillance endoscopy before and after sleeve gastrectomy? World J Gastrointest Endosc 11:1–4. 10.4253/wjge.v11.i1.1 30705727 10.4253/wjge.v11.i1.1 PMC6354110

[27] Giuliani S, Decker E, Leva E, Riccipetitoni G, Bagolan P (2016) Long term follow-up and transition of care in anorectal malformations: an international survey. J Pediatr Surg 51:1450–1457. 10.1016/j.jpedsurg.2016.03.011 27114308 10.1016/j.jpedsurg.2016.03.011

[28] Davies MC, Liao L-M, Wilcox DT, Woodhouse CRJ, Creighton SM (2010) Anorectal malformations: what happens in adulthood? BJU Int 106:398–404. 10.1111/j.1464-410X.2009.09031.x 19888969 10.1111/j.1464-410X.2009.09031.x

[29] Ludman L, Spitz L, Kiely EM (1994) Social and emotional impact of faecal incontinence after surgery for anorectal abnormalities. Arch Dis Child 71:194–200. 10.1136/adc.71.3.194 7979490 10.1136/adc.71.3.194 PMC1029970

[30] Violani C, Grano C, Fernandes M, Prato AP, Feitz WFJ, Wijnen R, Battye M, Schwarzer N, Lemli A, Cavalieri D, Aminoff D (2022) The transition of care for patients with anorectal malformations and Hirschsprung disease: a European survey. Eur J Pediatr Surg 33:191–197. 10.1055/s-0042-1749212 35830861 10.1055/s-0042-1749212

[31] Cairo SB, Gasior A, Rollins MD, Rothstein DH (2018) Challenges in transition of care for patients with anorectal malformations: a systematic review and recommendations for comprehensive care. Dis Colon Rectum 61:390–399. 10.1097/DCR.0000000000001033 29420431 10.1097/DCR.0000000000001033

[32] Goodhand J, Hedin CR, Croft NM, Lindsay JO (2011) Adolescents with IBD: the importance of structured transition care. J Crohns Colitis 5:509–519. 10.1016/j.crohns.2011.03.015 22115368 10.1016/j.crohns.2011.03.015

[33] Chan P, McNamara J, Vernon-Roberts A, Giles EM, Havrlant R, Christensen B, Thomas A, Williams A-J (2025) Systematic review: practices and programs in inflammatory bowel disease transition care. Inflamm Bowel Dis 31:1404–1418. 10.1093/ibd/izae190 39197100 10.1093/ibd/izae190

[34] Choi BH, Cohen D, Kitchens C, Schwartzberg DM (2025) Management of J-pouch complications. Surg Clin North Am 105:357–373. 10.1016/j.suc.2024.10.002 40015821 10.1016/j.suc.2024.10.002

[35] Ladd AP, Grosfeld JL (2006) Gastrointestinal tumors in children and adolescents. Semin Pediatr Surg 15:37–47. 10.1053/j.sempedsurg.2005.11.007 16458845 10.1053/j.sempedsurg.2005.11.007

[36] Hudson MM, Ness KK, Gurney JG, Mulrooney DA, Chemaitilly W, Krull KR, Green DM, Armstrong GT, Nottage KA, Jones KE, Sklar CA, Srivastava DK, Robison LL (2013) Clinical ascertainment of health outcomes among adults treated for childhood cancer. JAMA 309:2371. 10.1001/jama.2013.6296 23757085 10.1001/jama.2013.6296 PMC3771083

[37] Children’s Oncology Group (2018) Long-term follow-up guidelines for survivors of childhood, adolescent, and young adult cancers, version 5.0 10.1001/jamaoncol.2024.6812 PMC12188901 39976936

[38] Mertens AC, Liu Q, Neglia JP, Wasilewski K, Leisenring W, Armstrong GT, Robison LL, Yasui Y (2008) Cause-specific late mortality among 5-year survivors of childhood cancer: the Childhood Cancer Survivor Study. J Natl Cancer Inst 100:1368–1379. 10.1093/jnci/djn310 18812549 10.1093/jnci/djn310 PMC2556702

[39] Stefanowicz M, Kaliciński P, Ismail H, Kowalski A, Broniszczak D, Szymczak M, Pankowska-Woźniak K, Roszkiewicz A, Święszkowska E, Kamińska D, Szymańska S, Kowalewski G (2024) Risk for recurrence in long-term follow-up of children after liver transplantation for hepatoblastoma or hepatocellular carcinoma. Children 11:193. 10.3390/children11020193 38397305 10.3390/children11020193 PMC10887907

[40] Mulder RL, Font-Gonzalez A, Hudson MM, van Santen HM, Loeffen EAH, Burns KC, Quinn GP, van Dulmen-den Broeder E, Byrne J, Haupt R, Wallace WH, van den Heuvel-Eibrink MM, Anazodo A, Anderson RA, Barnbrock A, Beck JD, Bos AME, Demeestere I, Denzer C, Di Iorgi N, Hoefgen HR, Kebudi R, Lambalk C, Langer T, Meacham LR, Rodriguez-Wallberg K, Stern C, Stutz-Grunder E, van Dorp W, Veening M, Veldkamp S, van der Meulen E, Constine LS, Kenney LB, van de Wetering MD, Kremer LCM, Levine J, Tissing WJE, PanCareLIFE Consortium (2021) Fertility preservation for female patients with childhood, adolescent, and young adult cancer: recommendations from the PanCareLIFE Consortium and the International Late Effects of Childhood Cancer Guideline Harmonization Group. Lancet Oncol 22 e45–e56 10.1016/S1470-2045(20)30594-5 10.1016/S1470-2045(20)30594-5 33539753

