# Supplementary material for: Handing off hope: transition of care in pediatric surgery
Source: Surg Endosc. 2026 Mar 2;40(4):2705–12. doi: 10.1007/s00464-025-12561-z (PMC13053366; doi:10.1007/s00464-025-12561-z)
Supplement: Supplementary file 1 — Supplementary material 1 (PDF 511.5 kb) [file 464_2025_12561_MOESM1_ESM.pdf]

Patient Transition Readiness Assessment

Date: ____________________

Name:____________________________ Date of birth:______________________

Transition Importance and Confidence:

Please check box next to your response on a scale of 0-10
(0=not confident at all, 10=completely confident)

How important is it to you to manage your own health care?

☐ 1 ☐ 2 ☐ 3 ☐ 4 ☐ 5 ☐ 6 ☐ 7 ☐ 8 ☐ 9 ☐ 10

How important is it for you to transfer to adult doctors before age 22?

☐ 1 ☐ 2 ☐ 3 ☐ 4 ☐ 5 ☐ 6 ☐ 7 ☐ 8 ☐ 9 ☐ 10

How confident do you feel about your ability to transfer to an adult doctor?

☐ 1 ☐ 2 ☐ 3 ☐ 4 ☐ 5 ☐ 6 ☐ 7 ☐ 8 ☐ 9 ☐ 10

My Health:	Yes, I know this	I need to work on this	Who can help with this?
I know my medical needs.	☐	☐	
I can explain my medical needs to others.	☐	☐	
I know my symptoms including those that require me to see a doctor quickly.	☐	☐	
I know what to do in case of an emergency.	☐	☐	
I know my medicines, why I take them, and when to take them.	☐	☐	
I know my allergies and what medicines I should not take.	☐	☐	
I have and carry important medical information daily (health insurance, emergency contact, allergies, medications, health summary).	☐	☐	
I know how consent and health care privacy change when I am 18 (legal adult).	☐	☐	
I can explain my customs and beliefs to others and how they affect my health care treatment and decisions.	☐	☐	

Using Health Care:	Yes, I know this	I need to work on this	Who can help with this?
I can find my doctor's number.	☐	☐	
I can make my own doctor appointments.	☐	☐	
Before my visit, I think of questions to ask.	☐	☐	
I have transportation to my doctor's office.	☐	☐	
I know to show up 15 minutes before an appointment.	☐	☐	
I know where to get medical care when my doctor's office is closed.	☐	☐	
I have a file at home with my medical information.	☐	☐	
I have a copy of my current plan of care.	☐	☐	
I can fill out medical forms.	☐	☐	

(Continued on back)

Using Health Care(continued):	Yes, I know this	I need to work on this	Who can help with this?
I know how to get referrals to other providers.	☐	☐	
I have a pharmacy and know how to refill my medications.	☐	☐	
I know where to obtain tests (x-rays/labs) if my doctor orders them.	☐	☐	
I have a plan to keep health insurance after I turn 18 or older.	☐	☐	
My family and I have discussed my ability to make my own health care decisions at age 18.	☐	☐	
