# Supplementary material for: Handing off hope: transition of care in pediatric surgery
Source: Surg Endosc. 2026 Mar 2;40(4):2705–12. doi: 10.1007/s00464-025-12561-z (PMC13053366; doi:10.1007/s00464-025-12561-z)
Supplement: Supplementary file 2 — Supplementary material 2 (PDF 582.6 kb) [file 464_2025_12561_MOESM2_ESM.pdf]

Caregiver Transition Readiness Assessment

Date: ___________________

Name:__________________________ Date of birth:_____________________

Transition Importance and Confidence:

Please check box next to your response on a scale of 0-10
(0=not confident at all, 10=completely confident)

How important is it for your child to transfer to adult doctors before age 22

☐ 1 ☐ 2 ☐ 3 ☐ 4 ☐ 5 ☐ 6 ☐ 7 ☐ 8 ☐ 9 ☐ 10

How confident do you feel about your child's ability to transfer to an adult doctor?

☐ 1 ☐ 2 ☐ 3 ☐ 4 ☐ 5 ☐ 6 ☐ 7 ☐ 8 ☐ 9 ☐ 10

My health:	Yes, I know this	I need to work on this	Who can help with this?
My child knows his/her medical needs.	☐	☐	
My child can explain his/her medical needs to others.	☐	☐	
My child knows his/her symptoms including those that require a doctor quickly.	☐	☐	
My child knows what to do in case of an emergency.	☐	☐	
My child knows his/her medicines, why he/she takes them, and when to take them.	☐	☐	
My child knows his/her allergies and what medicines he/she should not take.	☐	☐	
My child carries important medical information daily (health insurance, emergency contact, allergies, medications, health summary).	☐	☐	
My child knows how consent and health care privacy change when he/she turns 18 (legal adult).	☐	☐	
My child can explain his/her customs and beliefs to others and how they affect his/her health care treatment and decisions.	☐	☐	

Using Health Care:	Yes, I know this	I need to work on this	Who can help with this?
My child can find his/her doctor's number.	☐	☐	
My child can make his/her own doctors appointments.	☐	☐	
Before his/her visit, my child thinks of questions to ask.	☐	☐	
My child has transportation to his/her doctor's office.	☐	☐	
My child knows to show up 15 minutes before an appointment.	☐	☐	
My child knows where to get medical care when the doctor's office is closed.	☐	☐	
My child has a file at home with his/her medical information.	☐	☐	
My child has a copy of his/her current plan of care.	☐	☐	

(Continued on back)

Using Health Care (continued):

	Yes, I know this	I need to work on this	Who can help with this?
My child can fill out medical forms.	☐	☐	
My child knows how to get referrals to other providers.	☐	☐	
My child has a pharmacy and knows how to refill medications.	☐	☐	
My child knows where to obtain tests (x-rays/labs) if the doctor orders them.	☐	☐	
My child has a plan to keep health insurance after he/she turns 18 or older.	☐	☐	
My child and I have discussed his/her ability to make his/her own health care decisions at age 18.	☐	☐	
My child and I have discussed a plan for supported decision making, if needed.	☐	☐	
